# Estimating Undercoverage Bias of Internet Users

**DOI:** 10.5888/pcd17.200026

**Published:** 2020-09-10

**Authors:** Jason Hsia, Guixiang Zhao, Machell Town

**Affiliations:** 1 Division of Population Health, Centers for Disease Control and Prevention, Atlanta, Georgia

## Abstract

**Introduction:**

In the last decade, response rates to the Behavioral Risk Factor Surveillance System (BRFSS) surveys have been declining. Attention has turned to the possibility of using web surveys to complement or replace BRFSS, but web surveys can introduce coverage bias as a result of excluding noninternet users. The objective of this study was to describe undercoverage bias of internet use.

**Methods:**

We used data from 402,578 respondents who completed BRFSS questions in 2017 on internet use, self-reported health, current smoking, and binge drinking. We examined undercoverage bias of internet use by partitioning it into a product of 2 components: proportion of noninternet use and difference in the prevalences of interest (self-reported health, current smoking, and binge drinking) between internet users and noninternet users.

**Results:**

Overall, the weighted proportion of noninternet use overall was 15.0%; the proportion increased with an increase in age and a decrease in education and, by race/ethnicity, was lowest among non-Hispanic white respondents. The overall relative bias was −19.2% for self-reported health, −4.0% for current cigarette smoking, and 8.4% for binge drinking. For all 3 variables of interest, we found large biases and relative biases in some demographic subgroups.

**Conclusion:**

Undercoverage bias of internet use existed in the 3 studied variables. Both proportion of noninternet users and difference in prevalences of studied variables between internet users and noninternet users contributed to the bias to different degrees. These findings have implications on helping health-related behavioral risk factor surveys transition to more cost-effective survey modes than telephone only.

**Summary**

**What is already known on this topic?**

Use of web surveys on health-related behavioral risk factors is increasing. Web surveys can save costs compared with face-to-face and telephone surveys, but they can introduce coverage bias as a result of excluding noninternet users.

**What is added by this report?**

We estimated the undercoverage bias that would result if only internet users participated in the Behavioral Risk Factor Surveillance System. We detected different levels of undercoverage bias for our 3 variables of interest: self-reported health, current smoking, and binge drinking.

**What are the implications for public health practice?**

Knowledge of undercoverage biases can be used in designing web surveys that assess health-related behavioral risk factors similar to BRFSS, specifically in selection of representative samples and sample size allocation of subpopulation groups.

## Introduction

Each year, the Behavioral Risk Factor Surveillance System (BRFSS), the world’s largest continuously run telephone-based health survey, collects data on participants’ health, use of preventive services, health care access, and health-related behavioral risk factors, such as tobacco and alcohol use, sedentary lifestyle/regular physical activity, and other behaviors in 50 states, the District of Columbia, and participating US territories (1). The target population of BRFSS is noninstitutionalized US residents aged 18 years or older. As found in many other surveys, response rates of BRFSS (2) have been decreasing, and the costs of maintaining an adequate response rate have been increasing correspondingly, leading to high costs.

Web surveys save field operational costs, which is a major advantage over traditional telephone or face-to-face interviews. Their common disadvantage, because they are likely voluntary samples, is that they underrepresent some subgroups in a target population, which presents a challenge for estimating characteristics of the target population. Because internet use has become more prevalent in recent years (3,4), the application of web surveys to BRFSS could be an alternative to the current mode of data collection. For example, one could use telephone surveys to select a probability sample and send a link to potential respondents to participate in a web survey or conduct a telephone survey (as BRFSS has been doing) and switch to a web survey when a potential respondent discontinues the telephone survey. Not everyone uses the internet, however, and little is known about the degree of bias caused by noninternet use in surveillance of general health and health-related behavioral risk factors. The 2017 BRFSS included a question about internet use. In this study, we estimated undercoverage bias by using the information on internet use collected from an aggregated state-representative sample.

## Methods

Data used in this study are from the 2017 BRFSS and were aggregated from all 50 states and the District of Columbia. A total of 450,016 eligible people completed the telephone interviews. We used binary answers (yes or no) to the question “Have you used the internet in the past 30 days?” to differentiate between internet users and noninternet users. We studied 3 outcome variables in BRFSS: self-reported fair or poor health, current cigarette smoking, and binge drinking of alcoholic beverages. Survey participants’ self-rated health was dichotomized into fair or poor and good/very good/excellent. Current smoking was defined as participants who had smoked at least 100 cigarettes during their lifetime and were still smoking at the time the survey was conducted. Binge drinking was defined as participants who had an episode of consuming at least 5 drinks (for men) or 4 drinks (women) on 1 occasion during the previous 30 days. In this study, we used 402,578 observations that had complete information on the 4 variables: internet use, self-reported fair or poor health, current cigarette smoking, and binge drinking.

To assess the coverage bias, we assumed that all internet users in the past 30 days would participate in a web survey of similar content and that the response rate would be 100%. Furthermore, let *P*, *P*_I_, and *P*_NI_ be the prevalence of an outcome variable among adults overall, among internet users only, and among noninternet users only, respectively; let *N* and *N*_NI_ be the total number and number of noninternet users, respectively. We used

$B=P_{I}-P=\frac{N_{NI}}{N}\left(P_{I}-P_{NI}\right)$

to assess the bias of undercoverage (*B*) when only internet users were included (ie, the difference between prevalences for internet users and for both internet users and noninternet users). In addition, we used relative bias (*RB*)

$RB=\frac{P_{I}-P}{P}$

to measure the effect of web survey on undercoverage. The estimation of bias ($\hat{B}$) and relative bias ($\hat{RB}$) were based on a correspondent formula of the sample:

$\hat{B}=p_{I}-p=\frac{n_{NI}}{n}\left(p_{I}-p_{NI}\right)$ and $\hat{RB}=\frac{p_{I}-p}{p}$

where lowercase letters represent the statistics that estimate correspondent parameters using the BRFSS sample and correspondent sample sizes. All estimates of prevalences, proportions, biases, and relative biases were weighted. Variances of these statistics were estimated by using Taylor linearization methods while taking complex survey design into account.

## Results

Among 402,578 respondents in the 2017 BRFSS, 15.0% reported not using the internet in the 30 days before the interview. The proportion of noninternet users increased with an increase in age (Table 1): it was lowest (3.9%) in the group aged 18 to 34 and highest (49.6%) in the group aged 75 or older. We found the highest proportion of the noninternet users among respondents with the lowest level of education (<high school diploma, 45.3%) and the lowest level of household income (<100% federal poverty level, 29.7%) and, by race/ethnicity, among Hispanic (23.7%) and non-Hispanic black (22.0%) respondents.

**Table 1:** Demographic Characteristics of Sample by Internet Users and Proportion of Noninternet Users, BRFSS, 2017^a^

Characteristic	Total No.	No. of Internet Users	Noninternet Users	
			No.	Weighted % (95% CI)
**Overall**	402,578	331,790	70,788	15.0 (14.7–15.2)
**Age, y**				
18–34	64,980	62,894	2,086	3.9 (3.6–4.2)
35–44	46,326	43,776	2,550	7.3 (6.8–7.8)
45–54	62,036	55,762	6,274	12.1 (11.6–12.7)
55–64	87,185	73,108	14,077	19.3 (18.7–20.0)
65–74	83,878	64,926	18,952	26.4 (25.7–27.1)
≥75	58,173	31,324	26,849	49.6 (48.6–50.7)
**Sex**				
Men	176,670	147,183	29,487	14.6 (14.3–15.0)
Women	225,908	184,607	41,301	15.4 (15.0–15.7)
**Race/ethnicity**				
Non-Hispanic white	314,884	265,456	49,428	12.4 (12.2–12.6)
Non-Hispanic black	31,766	22,446	9,320	22.0 (21.2–22.9)
Hispanic	28,742	21,954	6,788	23.7 (22.7–24.7)
Non-Hispanic other	27,186	21,934	5,252	9.4 (8.7–10.1)
**Education**				
<High school diploma	27,442	12,452	14,990	45.3 (44.1–46.4)
High school diploma	108,531	76,443	32,088	20.4 (20.0–20.9)
Some college	112,595	97,403	15,192	8.3 (7.97–8.6)
≥College	154,010	145,492	8,518	3.2 (3.0–3.4)
**Living below federal poverty level (FPL)**				
<100% FPL	32,613	21,824	10,789	29.7 (28.6–30.7)
100%–199% FPL	73,058	50,392	22,666	25.3 (24.6–25.9)
≥200% FPL	196,564	179,844	16,720	6.4 (6.1–6.6)
Unknown	100,343	79,730	20,613	16.5 (16.0–16.9)

Abbreviation: BRFSS, Behavioral Risk Factor Surveillance System.

a Data are from 402,578 respondents in all 50 states and the District of Columbia who completed BRFSS questions in 2017 on internet use, self-reported health, current smoking, and alcohol use.

Overall, the difference in prevalence between internet users and noninternet users was −23.4% for self-reported fair or poor health (Table 2), −4.3% for current cigarette smoking (Table 3), and 9.6% for binge drinking (Table 4). For self-reported fair or poor health, the largest absolute differences in the prevalence between internet users and noninternet users were among those aged 45 to 54 (−25.3%), aged 55 to 64 (−27.1%), and Hispanic (−25.9%), and the prevalences were lower among internet users than noninternet users in all demographic subgroups. For current cigarette smoking, the pattern of the differences of prevalence by age groups was similar to that for self-reported health, but we also observed larger absolute differences among men (−7.8%), non-Hispanic black respondents (−7.4%), and non-Hispanic other respondents (−9.3%). Among those who had less than a high school education, the prevalence of current cigarette smoking was higher among internet users than noninternet users (30.5% vs 22.3%). For binge drinking, we found large differences between internet users and noninternet users among respondents aged 18 to 34 (12.6%) and 35 to 44 years (6.5%), non-Hispanic white respondents (11.4%), Hispanic respondents (10.7%), and those with at least some college (>10%).

**Table 2:** Prevalence of Self-Reported Fair or Poor Health, by Internet Use, Difference in Prevalence, Bias, and Relative Bias, by Demographic Characteristics, BRFSS, 2017^a^

Characteristic	% (95% CI)					
	All Adults [*P*]	Internet Users [*P*_I_]	Noninternet Users [*P*_NI_]	Difference [*P*_I_ − *P*_NI_]	Bias of Undercoverage When Only Internet Users Were Included [*P*_I_ – *P*]	Relative Bias [(*P*_I_ – *P*)/*P*]
**Overall**	18.3 (18.0 to 18.6)	14.8 (14.5 to 15.1)	38.2 (37.4 to 39.0)	−23.4 (−24.3 to −22.6)	−3.5 (−3.8 to −3.2)	−19.2 (−20.6 to −17.8)
**Age, y**						
18–34	10.8 (10.4 to 11.3)	10.5 (10.0 to 10.9)	19.8 (17.0 to 23.0)	−9.3 (−12.4 to −6.3)	−0.4 (−0.8 to 0.1)	−3.3 (−7.6 to 1.0)
35–44	15.4 (14.7 to 16.1)	14.1 (13.4 to 14.8)	32.5 (29.3 to 35.8)	−18.4 (−21.8 to −15.1)	−1.6 (−2.1 to −0.6)	−8.8 (−13.3 to −4.2)
45–54	20.0 (19.4 to 20.7)	17.0 (16.3 to 17.6)	42.3 (39.8 to 44.9)	−25.3 (−28.0 to −22.7)	−3.1 (−3. 8 to −2.4)	−15.3 (−18.6 to −12.0)
55–64	24.1 (23.5 to 24.8)	18.9 (18.2 to 19.6)	46.0 (44.2 to 47.9)	−27.1 (−29.1 to −25.2)	−5.2 (−6.0 to −4.5)	−21.7 (−24.4 to −19.1)
65–74	23.4 (22.7 to 24.1)	17.5 (16.8 to 18.2)	39.8 (38.2 to 41.4)	−22.3 (−24.1 to −20.6)	−5.9 (−6.6 to −5.1)	−25.2 (−28.1 to −22.3)
≥75	27.8 (26.9 to 28.7)	20.1 (19.0 to 21.2)	35.6 (34.2 to 37.1)	−15.5 (−17.3 to −13.7)	−7.7 (−8.8 to −6.6)	−27.7 (−31.4 to −24.1)
**Sex**						
Men	17.3 (16.9 to 17.6)	13.9 (13.5 to 14.3)	37.0 (35.8 to 38.3)	−23.1 (−24.4 to −21.9)	−3.4 (−3.8 to −3.0)	−19.6 (−21.7 to −17.5)
Women	19.3 (18.9 to 19.6)	15.6 (15.3 to 16.0)	39.3 (38.1 to 40.4)	−23.6 (−24.8 to −22.4)	−3.6 (−4.0 to −3.2)	−18.8 (−20.7 to −16.9)
**Race/ethnicity**						
Non-Hispanic white	16.4 (16.1 to 16.7)	13.7 (13.5 to 14.0)	35.2 (34.3 to 36.1)	−21.5 (−22.4 to −20.6)	−2.7 (−2.9 to −2.4)	−16.2 (−17.8 to −14.6)
Non-Hispanic black	22.1 (21.3 to 23.0)	17.4 (16.6 to 18.4)	38.8 (36.8 to 40.8)	−21.3 (−23.5 to −19.1)	−4.7 (−5.6 to −3.7)	−21.2 (−25.1 to −17.3)
Hispanic	25.1 (24.2 to 26.1)	19.0 (18.0 to 20.0)	44.9 (42.6 to 47.2)	−25.9 (−28.4 to −23.4)	−6.1 (−7.2 to −5.1)	−24.4 (−28.2 to −20.7)
Non-Hispanic other	15.1 (14.0 to 16.2)	12.9 (11.9 to 14.1)	35.5 (32.2 to 39.0)	−22.6 (−26.2 to −19.0)	−2.1 (−3.3 to −1.0)	−14.1 (−21.3 to −6.8)
**Education**						
<High school diploma	39.7 (38.6 to 40.9)	33.3 (31.8 to 34.8)	47.6 (45.9 to 49.2)	−14.3 (−16.5 to −12.0)	−6.5 (−7.9 to −5.0)	−16.2 (−19.7 to −12.7)
High school diploma	21.3 (20.8 to 21.8)	18.1 (17.6 to 18.7)	33.7 (32.6 to 34.8)	−15.6 (−16.8 to −14.3)	−3.2 (−3.7 to −2.6)	−14.9 (−17.5 to −12.4)
Some college	16.1 (15.7 to 16.5)	14.7 (14.2 to 15.1)	32.0 (30.3 to 33.7)	−17.3 (−19.1 to −15.5)	−1.4 (−1.9 to −1.0)	−8.9 (−11.7 to −6.0)
≥College	7.9 (7.7 to 8.2)	7.4 (7.1 to 7.7)	24.2 (22.2 to 26.4)	−16.8 (−19.0 to −14.7)	−0.5 (−0.8 to −0.2)	−6.8 (−10.4 to −3.2)
**Living below federal poverty level (FPL)**						
<100% FPL	35.0 (33.9 to 36.1)	30.1 (28.8 to 31.3)	46.5 (44.3 to 48.7)	−16.4 (−18.9 to −13.9)	−4.9 (−6.1 to −3.6)	−13.9 (−17.4 to −10.5)
100%–199% FPL	26.8 (26.1 to 27.6)	22.6 (21.8 to 23.4)	39.4 (38.0 to 40.9)	−16.9 (−18.5 to −15.2)	−4.3 (−5.1 to −3.4)	−15.9 (−18.8 to −13.0)
≥200% FPL	10.4 (10.1 to 10.7)	9.4 (9.1 to 9.7)	24.6 (23.1 to 26.1)	−15.2 (−16.7 to −13.7)	−1.0 (−1.3 to −0.6)	−9.3 (−12.3 to −6.3)
Unknown	19.0 (18.5 to 19.5)	14.9 (14.5 to 15.4)	39.3 (37.8 to 40.8)	−24.4 (−26.0 to −22.8)	−4.0 (−4.5 to −3.5)	−21.2 (−23.7 to −18.6)

Abbreviation: BRFSS, Behavioral Risk Factor Surveillance System.

a Data are from 402,578 respondents in all 50 states and the District of Columbia who completed BRFSS questions in 2017 on internet use, self-reported health, current smoking, and alcohol use.

**Table 3:** Prevalence of Current Cigarette Smoking, by Internet Use, Difference in Prevalences, Bias, and Relative Bias, by Demographic Characteristics, BRFSS, 2017^a^

Characteristic	% (95% CI)					
	All Adults [*P*]	Internet Users [*P*_I_]	Noninternet Users [*P*_NI_]	Difference [*P*_I_ − *P*_NI_]	Bias of Undercoverage When Only Internet Users Were Included [*P*_I_ – *P*]	Relative Bias [(*P*_I_ – *P*)/*P*]
**Overall**	16.1 (15.8 to 16.3)	15.4 (15.2 to 15.7)	19.7 (19.1 to 20.4)	−4.3 (−5.1 to −3.6)	−0.7 (−0.9 to −0.4)	−4.0 (−5.7 to −2.4)
**Age, y**						
18–34	17.1 (16.6 to 17.6)	17.0 (16.5 to 17.5)	18.9 (16.0 to 22.2)	−1.9 (−5.0 to 1.2)	−0.1 (−0.6 to 0.5)	−0.4 (−3.6 to 2.7)
35–44	19.9 (19.2 to 20.7)	19.5 (18.7 to 20.3)	25.6 (22.5 to 28.9)	−6.1 (−9.4 to −2.8)	−0.5 (−1.2 to 0.3)	−2.2 (−6.1 to 1.6)
45–54	18.1 (17.5 to 18.7)	16.6 (15.9 to 17.2)	29.3 (27.2 to 31.5)	−12.7 (−15.0 to −10.5)	−1.5 (−2.2 to −0.9)	−8.5 (−11.9 to −5.2)
55–64	17.6 (17.0 to 18.1)	14.5 (13.9 to 15.0)	30.5 (28.9 to 32.2)	−16.1 (−17.8 to −14.3)	−3.1 (−3.7 to −2.5)	−17.7 (−20.6 to −14.7)
65–74	11.2 (10.7 to 11.7)	8.7 (8.2 to 9.2)	18.2 (17.1 to 19.4)	−9.6 (−10.8 to −8.3)	−2.5 (−3.0 to −2.0)	−22.5 (−26.4 to −18.6)
≥75	5.1 (4.7 to 5.6)	3.8 (3.4 to 4.3)	6.5 (5.9 to 7.2)	−2.7 (−3.5 to −1.9)	−1.3 (−1.8 to −0.9)	−26.0 (−34.1 to −17.9)
**Sex**						
Men	18.2 (17.8 to 18.6)	17.0 (16.6 to 17.5)	24.8 (23.8 to 26.0)	−7.8 (−9.0 to −6.7)	−1.1 (−1.6 to −0.7)	−6.3 (−8.5 to −4.1)
Women	14.1 (13.8 to 14.4)	13.9 (13.6 to 14.2)	15.2 (14.4 to 16.0)	−1.3 (−2.2 to −0.4)	−0.2 (−0.5 to 0.1)	−1.4 (−3.8 to 0.95)
**Race/ethnicity**						
Non-Hispanic white	16.8 (16.5 to 17.1)	16.2 (15.9 to 16.5)	21.5 (20.7 to 22.3)	−5.4 (−6.2 to −4.5)	−0.7 (−1.0 to −0.4)	−4.0 (−5.7 to −2.2)
Non-Hispanic black	17.8 (17.0 to 18.6)	16.2 (15.3 to 17.1)	23.6 (21.8 to 25.4)	−7.4 (−9.4 to −5.4)	−1.6 (−2.5 to −0.8)	−9.2 (−13.9 to −4.5)
Hispanic	13.1 (12.3 to 13.9)	13.2 (12.3 to 14.1)	12.7 (11.2 to 14.4)	0.5 (−1.4 to 2.3)	0.1 (−0.7 to 1.0)	0.8 (−5.8 to 7.5)
Non-Hispanic other	13.4 (12.5 to 14.3)	12.5 (11.6 to 13.5)	21.8 (19.3 to 24.6)	−9.3 (−12.1 to −6.5)	−0.9 (−1.8 to 0.1)	−6.5 (−13.4 to 0.4)
**Education**						
<High school diploma	26.7 (25.8 to 27.8)	30.5 (29.0 to 32.0)	22.3 (21.0 to 23.6)	8.2 (6.3 to 10.2)	3.7 (2.4 to 5.1)	13.9 (8.8 to 19.0)
High school diploma	20.7 (20.2 to 21.2)	21.1 (20.5 to 21.7)	19.3 (18.4 to 20.2)	1.8 (0.7 to 2.8)	0.4 (−0.2 to 0.9)	1.7 (−0.97 to 4.5)
Some college	16.3 (15.9 to 16.8)	16.1 (15.6 to 16.6)	18.5 (17.1 to 20.1)	−2.4 (−4.0 to −0.8)	−0.2 (−0.7 to 0.3)	−1.2 (−4.2 to 1.7)
≥College	6.3 (6.1 to 6.5)	6.2 (5.9 to 6.4)	9.8 (8.4 to 11.5)	−3.7 (−5.2 to −2.2)	−0.1 (−0.4 to 0.1)	−1.9 (−5.7 to 1.96)
**Living below federal poverty level (FPL)**						
<100% FPL	25.2 (24.2 to 26.1)	25.9 (24.8 to 27.0)	23.5 (21.8 to 25.3)	2.4 (0.3 to 4.5)	0.7 (−0.4 to 1.7)	2.8 (−1.5 to 7.1)
100%–199% FPL	21.2 (20.5 to 21.8)	21.5 (20.7 to 22.3)	20.1 (18.9 to 21.3)	1.4 (0 to 2.9)	0.4 (−0.4 to 1.1)	1.7 (−1.9 to 5.3)
≥200% FPL	11.7 (11.4 to 12.1)	11.5 (11.1 to 11.8)	15.5 (14.3 to 16.8)	−4.1 (−5.3 to −2.8)	−0.3 (−0.6 to 0.1)	−2.2 (−5.1 to 0.7)
Unknown	16.2 (15.7 to 16.7)	15.6 (15.1 to 16.1)	19.3 (18.2 to 20.5)	−3.7 (−5.0 to −2.5)	−0.6 (−1.1 to −0.1)	−3.8 (−6.8 to −0.7)

Abbreviation: BRFSS, Behavioral Risk Factor Surveillance System.

a Data are from 402,578 respondents in all 50 states and the District of Columbia who completed BRFSS questions in 2017 on internet use, self-reported health, current smoking, and alcohol use.

**Table 4:** Prevalence of Binge Drinking, by Internet Use, Difference in Prevalence, Bias, and Relative Bias, by Demographic Characteristics, BRFSS, 2017^a^

Characteristic	% (95% CI)					
	All Adults [*P*]	Internet Users [*P*_I_]	Noninternet Users [*P*_NI_]	Difference [*P*_I_ − *P*_NI_]	Bias of Undercoverage When Only Internet Users Were Included [*P*_I_ – *P*]	Relative Bias [(*P*_I_ – *P*)/*P*]
**Overall**	17.1 (16.9 to 17.4)	18.6 (18.3 to 18.8)	9.0 (8.5 to 9.5)	9.6 (9.0 to 10.2)	1.4 (1.1 to 1.7)	8.4 (6.6 to 10.2)
**Age, y**						
18–34	26.5 (25.8 to 27.1)	26.9 (26.3 to 27.6)	14.3 (11.8 to 17.2)	12.6 (9.9 to 15.4)	0.5 (−0.2 to 1.1)	1.8 (−0.6 to 4.3)
35–44	20.8 (20.1 to 21.6)	21.3 (20.5 to 22.1)	14.8 (12.4 to 17.6)	6.5 (3.7 to 9.2)	0.5 (−0.3 to 1.3)	2.3 (−1.5 to 6.1)
45–54	17.1 (16.5 to 17.8)	17.3 (16.6 to 18.0)	16.0 (14.2 to 18.1)	1.3 (−0.8 to 3.3)	0.2 (−0.5 to 0.8)	0.9 (−3.1 to 4.9)
55–64	11.7 (11.2 to 12.2)	11.8 (11.3 to 12.4)	11.2 (10.2 to 12.3)	0.6 (−0.5 to 1.7)	0.1 (−0.4 to 0.6)	1.0 (−3.5 to 5.5)
65–74	6.9 (6.5 to 7.4)	6.9 (6.5 to 7.4)	6.9 (6.0 to 8.0)	0 (−1.1 to 1.1)	0 (−0.4 to 0.5)	0 (−6.4 to 6.5)
≥75	2.6 (2.3 to 2.9)	2.9 (2.5 to 3.3)	2.3 (1.9 to 2.7)	0.6 (0 to 1.2)	0.3 (−0.1 to 0.7)	12.2 (−3.2 to 27.6)
**Sex**						
Men	22.3 (21.9 to 22.7)	23.6 (23.1 to 24.1)	14.5 (13.6 to 15.5)	9.1 (8.0 to 10.1)	1.3 (0.9 to 1.8)	5.9 (3.8 to 8.1)
Women	12.3 (12.0 to 12.6)	13.8 (13.4 to 14.2)	4.0 (3.5 to 4.5)	9.8 (9.2 to 10.4)	1.5 (1.2 to 1.9)	12.2 (9.2 to 15.3)
**Race/ethnicity**						
Non-Hispanic white	17.8 (17.6 to 18.1)	19.3 (18.9 to 19.6)	7.8 (7.3 to 8.4)	11.4 (10.8 to 12.1)	1.4 (1.1 to 1.7)	7.9 (6.1 to 9.8)
Non-Hispanic black	13.4 (12.7 to 14.2)	14.4 (13.6 to 15.3)	9.9 (8.6 to 11.4)	4.5 (2.9 to 6.1)	1.0 (0.1 to 1.8)	7.4 (0.8 to 14.0)
Hispanic	18.8 (17.9 to 19.7)	21.3 (20.2 to 22.4)	10.7 (9.3 to 12.2)	10.7 (8.8 to 12.5)	2.5 (1.5 to 3.6)	13.4 (7.4 to 19.5)
Non-Hispanic other	13.7 (12.7 to 14.7)	14.1 (13.0 to 15.2)	9.7 (7.8 to 12.0)	4.4 (2.0 to 6.7)	0.4 (−0.7 to 1.5)	3.0 (−5.1 to 11.0)
**Education**						
<High school diploma	13.6 (12.8 to 14.5)	17.6 (16.4 to 18.8)	8.8 (7.9 to 9.8)	8.7 (7.2 to 10.3)	4.0 (2.8 to 5.1)	29.0 (20.0 to 38.0)
High school diploma	16.5 (16.0 to 17.0)	18.3 (17.7 to 18.9)	9.5 (8.7 to 10.2)	8.9 (7.9 to 9.8)	1.8 (1.2 to 2.4)	11.0 (7.3 to 14.7)
Some college	18.4 (17.9 to 18.9)	19.3 (18.7 to 19.8)	8.8 (7.7 to 10.0)	10.5 (9.3 to 11.8)	0.9 (0.3 to 1.4)	4.7 (1.6 to 7.8)
≥College	17.9 (17.5 to 18.3)	18.2 (17.8 to 18.7)	7.3 (5.8 to 9.1)	11.0 (9.2 to 12.7)	0.4 (−0.1 to 0.8)	2.0 (−0.5 to 4.4)
**Living below federal poverty level (FPL)**						
<100% FPL	14.9 (14.1 to 15.7)	16.9 (15.9 to 18.0)	10.1 (8.8 to 11.5)	6.8 (5.1 to 8.6)	2.0 (1.0 to 3.1)	13.7 (6.4 to 20.9)
100%–199% FPL	14.5 (13.9 to 15.1)	16.6 (15.8 to 17.3)	8.4 (7.5 to 9.4)	8.1 (6.9 to 9.3)	2.1 (1.3 to 2.8)	14.1 (8.8 to 19.5)
≥200% FPL	19.3 (18.9 to 19.7)	19.9 (19.5 to 20.3)	11.1 (9.8 to 12.4)	8.8 (7.5 to 10.2)	0.6 (0.1 to 1.0)	2.9 (0.6 to 5.2)
Unknown	16.1 (15.7 to 16.6)	17.9 (17.3 to 18.4)	7.4 (6.7 to 8.2)	10.4 (9.5 to 11.4)	1.7 (1.2 to 2.3)	10.7 (7.2 to 14.2)

Abbreviation: BRFSS, Behavioral Risk Factor Surveillance System.

a Data are from 402,578 respondents in all 50 states and the District of Columbia who completed BRFSS questions in 2017 on internet use, self-reported health, current smoking, and alcohol use.

The overall bias and relative bias were −3.5% and −19.2% for self-reported fair or poor health (Table 2), −0.7% and −4.0% for current smoking (Table 3), and 1.4% and 8.4% for binge drinking (Table 4). The bias varied by demographic characteristics. For self-reported fair or poor health, we found the largest bias and relative bias occurred among the groups aged 55 or older, non-Hispanic black and Hispanics respondents, those with a low level of education, and those with a low household income. For current smoking, the bias and relative bias had a similar pattern to that for self-reported fair or poor health. For binge drinking, the largest relative bias was found among respondents aged 75 or older, women, Hispanic respondents, those with less than a high school education, and those with low household income.

## Discussion

We found that undercoverage bias of internet use existed in the 3 studied variables. Both proportion of internet use and the differences in prevalences of studied variables between internet users and noninternet users contributed to the bias to different degrees.

Our study showed that the proportion of noninternet use increased as age group increased and education level decreased and was lowest, by racial/ethnic group, among non-Hispanic white respondents, which is consistent with a previous report (5). Undercoverage in a web survey would not be a problem if internet users did not differ systematically from noninternet users. We found differences, however, between internet users and noninternet users. Noninternet users, therefore, could not be considered a random sample from the target population, meaning that valid conclusions could not be drawn from the web survey.

We found 3 types of significant biases. First, both the proportion of noninternet users and the absolute difference in prevalence between internet users and noninternet users was large in some groups, for example, among the group aged 55 to 64 and the group with less than a high school diploma for self-reported fair or poor health and current cigarette smoking. Second, the proportion of noninternet users was large, but the difference in prevalence between internet users and noninternet users was small in some groups, for example, among adults aged 75 years or older for current smoking and binge drinking. Third, the proportion of noninternet users was small, but the difference in prevalence between internet users and noninternet users was large in some groups, for example, among adults aged 18 to 34 years for binge drinking. The findings among these subgroups suggest we need to examine both the proportion of noninternet users and the difference between internet users and noninternet users at the same time when we evaluate undercoverage bias, rather than focus on the proportion of noninternet users only (3).

Although bias indicates the difference between prevalence among the internet users only and overall prevalence, the relative biases, after rescaling the biases with respect to overall prevalence, reflect the strength and direction of the biases. Self-reported health is a key variable in health surveys such as BRFSS and the National Health Interview Survey. If only internet users were included in a probability sample, the prevalence of self-reported fair or poor health would be underestimated with a large negative relative bias, possibly because self-reported fair or poor health and not using the internet are associated with lower socioeconomic status. The relative bias for self-reported fair or poor health was much higher than for current cigarette smoking and binge drinking. For current cigarette smoking, another important indicator and a modifiable behavioral risk factor, the relative biases were similar to those for self-reported health among older participants but indicated overestimation rather than underestimation among those who had not completed high school. Binge drinking is another modifiable behavioral risk factor, but unlike for current smoking, the relative biases for binge drinking indicated overestimation for all sociodemographic subgroups. Because these findings could have a major effect on planning web health surveys, survey designers, samplers, and interviewers need to put more effort into including those subgroups.

Findings from this study could be useful for some types of surveys on health-related risk factors. For example, one could select a probability sample using a telephone survey mode and then provide a link to a web survey in a text message. Self-reported health and/or current smoking are often used as key variables in health-related risk factor surveys. The difference in the prevalences between internet use and noninternet use in some demographic groups indicate that studied internet users are not a random sample of a general population; therefore, study designers should pay particular attention when conducting calibrations in weighting. Our findings could help web survey designers recruit larger sample sizes in certain demographic groups. Underestimation of self-reported health and overestimation of binge drinking could also help analysts to interpret the web survey results. Another example would be a mixed mode survey. A participating state can conduct a much shorter version of BRFSS to increase the response rate and switch to a web survey to ask additional questions on smoking and drinking. In this scenario, our findings could be useful for bias corrections.

Our study has some limitations. First, it was based on a sample survey, BRFSS, instead of population-level data, such as a complete population registry. Second, BRFSS is a telephone survey; raking and other adjustments for a relatively large nonresponse rate added additional uncertainty (6). Third, we assumed that all adults who had used the internet during the previous month would respond to a web survey. This is generally not true in practice. Fourth, BRFSS nonrespondents likely also included internet users. Some people who decline to participate in a survey by telephone might agree to participate in a web-based format if they were reached and motivated (7). The inclusion of BRFSS nonrespondents in a cumulative sample of internet users might change our estimates in a general situation. Our study, however, examined a part of undercoverage biases.

The term “web survey” has so many meanings so that it conveys little information about how a survey is conducted (8). Our study was a preliminary study of undercoverage biases of internet use that used data from a large population-based survey on gauging self-reported health, current cigarette smoking, and binge drinking, 3 important measures in public health. We detected different levels of undercoverage biases for overall prevalence and subgroup prevalences. Both proportions of noninternet users and differences in prevalence between internet users and noninternet users play a role in assessing undercoverage biases. Our research can affect the design of web surveys intended to complement or replace the existing BRFSS. Because the applicability of a new survey mode for BRFSS is open, we need studies to collect new empirical evidence. Areas of future research could include the assessment of web survey response rates; the testing of mixed mode, mail/web, telephone/web, and other approaches to data collection and analysis; and the evaluation of the validity and reliability of future BRFSS questionnaire items with an external data source. As web surveys are increasingly used, the findings of our study and future research have implications for helping health and health-related behavioral risk factor surveys transition to more cost-effective survey modes.

## References

[1] Centers for Disease Control and Prevention. Behavioral Risk Factor Surveillance System. https://www.cdc.gov/brfss. Accessed December 19, 2019.

[2] Czajka JL , Beyler A . Declining response rates in federal surveys: trends and implications. June 15, 2016. https://www.mathematica.org/our-publications-and-findings/publications/declining-response-rates-in-federal-surveys-trends-and-implications-background-paper. Accessed December 19, 2019.

[3] Bethlehem J , Biffignandi S . Handbook of web surveys. Hoboken (NJ): John Wiley & Sons, Inc; 2012.

[4] Ryan C . Computer and internet use in the United States: 2016. American Community Survey Reports, ACS-39. Washington (DC): US Census Bureau; 2018. https://www.census.gov/content/dam/Census/library/publications/2018/acs/ACS-39.pdf. Accessed December 19, 2019.

[5] Cohen RA , Adams PF . Use of the internet for health information: United States, 2009. NCHS Data Brief 2011;(66):1–8. 22142942

[6] Centers for Disease Control and Prevention. Methodologic changes in the Behavioral Risk Factor Surveillance System in 2011 and potential effects on prevalence estimates. MMWR Morb Mortal Wkly Rep 2012;61(22):410–3. 22672976

[7] Edwards PJ , Roberts I , Clarke MJ , Diguiseppi C , Wentz R , Kwan I , Methods to increase response to postal and electronic questionnaires. Cochrane Database Syst Rev 2009;3(3):MR000008. 10.1002/14651858.MR000008.pub4 19588449 PMC8941848

[8] Couper MP , Miller PV . Web survey methods — introduction. Public Opin Q 2008;72(5):831–5. 10.1093/poq/nfn066

